# Short-lasting unilateral neuralgiform headache attacks with ispilateral facial flushing is a new variant of paroxysmal extreme pain disorder

**DOI:** 10.1186/s10194-015-0519-3

**Published:** 2015-04-23

**Authors:** Noboru Imai, Noriko Miyake, Yoshiaki Saito, Emiko Kobayashi, Masako Ikawa, Shinya Manaka, Masaaki Shiina, Kazuhiro Ogata, Naomichi Matsumoto

**Affiliations:** Department of Neurology, Japanese Red Cross Shizuoka Hospital, 8-2 Ohtemachi, Aoi-ku, Shizuoka, Shizuoka 420-0853 Japan; Department of Human Genetics, Yokohama City University Graduate School of Medicine, 3-9 Fukuura, Kanazawa-ku, Yokohama, 236-0004 Japan; Department of Child Neurology, National Center of Neurology and Psychiatry, Tokyo, Japan; Division of Child Neurology, Institute of Neurological Sciences, Faculty of Medicine, Tottori University, Yonago, Japan; Department of Oral Surgery, Shizuoka-Shimizu Municipal Hospital, Shizuoka, Japan; Department of Neurosurgery, Manaka Hospital, Kanagawa, Japan; Department of Biochemistry, Yokohama City University Graduate School of Medicine, Yokohama, Japan

**Keywords:** Unilateral headache, Facial flushing, *SCN9A* mutation, Paroxysmal extreme pain disorder, Voltage-gated sodium channel, Focal hyperperfusion

## Abstract

**Background:**

We encountered a 5-year-old girl who had short-lasting, severe, unilateral temporal headaches with ipsilateral lacrimation, nasal congestion and rhinorrhoea, and facial flushing after severe attacks. Family history revealed similar short-lasting, severe headaches in an older brother, younger sister, mother, maternal aunt, and maternal grandfather’s brother.

**Methods:**

We performed routine laboratory examinations and electrophysiological and radiological studies for three children, and whole-exome sequencing to determine the genetic causality in this family.

**Results:**

Focal hyperperfusion of the right trigeminal root entry zone was seen during a right-sided attack in one child, while left-sided temporal headache attacks were provoked by bilateral electrical stimulation of the upper extremities in another. We identified a novel *SCN9A* mutation (NM_002977: c.5218G>C, p.Val1740Leu) in all affected family members, but not in any of the unaffected members. *SCN9A* encodes the voltage-gated sodium-channel type IX alpha subunit known as Na_v_1.7.

**Conclusions:**

Gain-of-function mutations in Na_v_1.7 are well known to cause paroxysmal extreme pain disorder (PEPD), a painful Na-channelopathy characterized by attacks of excruciating deep burning pain in the rectal, ocular, or jaw areas. The *SCN9A* mutation suggests that our patients had a phenotype of PEPD with a predominant symptom of short-lasting, severe, unilateral headache.

**Electronic supplementary material:**

The online version of this article (doi:10.1186/s10194-015-0519-3) contains supplementary material, which is available to authorized users.

## Background

Paroxysmal extreme pain disorder (PEPD) was first described in 1959 as a familial disorder characterized by very brief episodes of excruciating rectal pain associated with flushing of the buttocks and legs, ocular pain and flushing of the eyelid and periorbital skin, and submaxillary pain [1]. In PEPD, autonomic symptoms of skin flushing, often with a harlequin pattern distribution, emerge during the neonatal period. Dramatic syncopes with bradycardia and sometimes asystole are also common. Attacks of excruciating deep burning pain in the rectal, ocular, or jaw areas emerge later in affected individuals. These attacks are triggered by factors such as defecation, cold wind, eating, and emotions. These painful symptoms are completely alleviated by carbamazepine in most patients [2].

*SCN9A* encodes the voltage-gated sodium-channel type IX alpha subunit known as Na_v_1.7, which is mainly expressed in peripheral sensory neurons of the dorsal root/sympathetic ganglia. Mutations in this gene can cause variable phenotypes with different patterns of inheritance. Gain-of-function mutations mainly cause primary erythermalgia [3], PEPD [2], small-fiber neuropathy [4], and chronic non-paroxysmal neuropathic pain [5] in an autosomal-dominant manner. *SCN9A* mutations also result in some features of Dravet syndrome [6] and febrile convulsions [7]. In addition, loss-of-function mutations in *SCN9A* can lead to indifference to pain [8,9] and hereditary sensory and autonomic neuropathy type IID [10] with autosomal-recessive inheritance.

Here, we describe familial cases of a painful disorder showing some clinical features reminiscent of those in PEPD, but with short-lasting, severe, unilateral headache over the cranium as the prominent symptom in most affected family members. We identified a novel *SCN9A* mutation in this family, suggesting that the family suffers from a variant of PEPD. Detailed clinical information of the proband and her siblings, and genetic data of her family, including the results of whole-exome sequencing (WES), are presented.

## Case reports

### Patient 1

**Figure 1:**
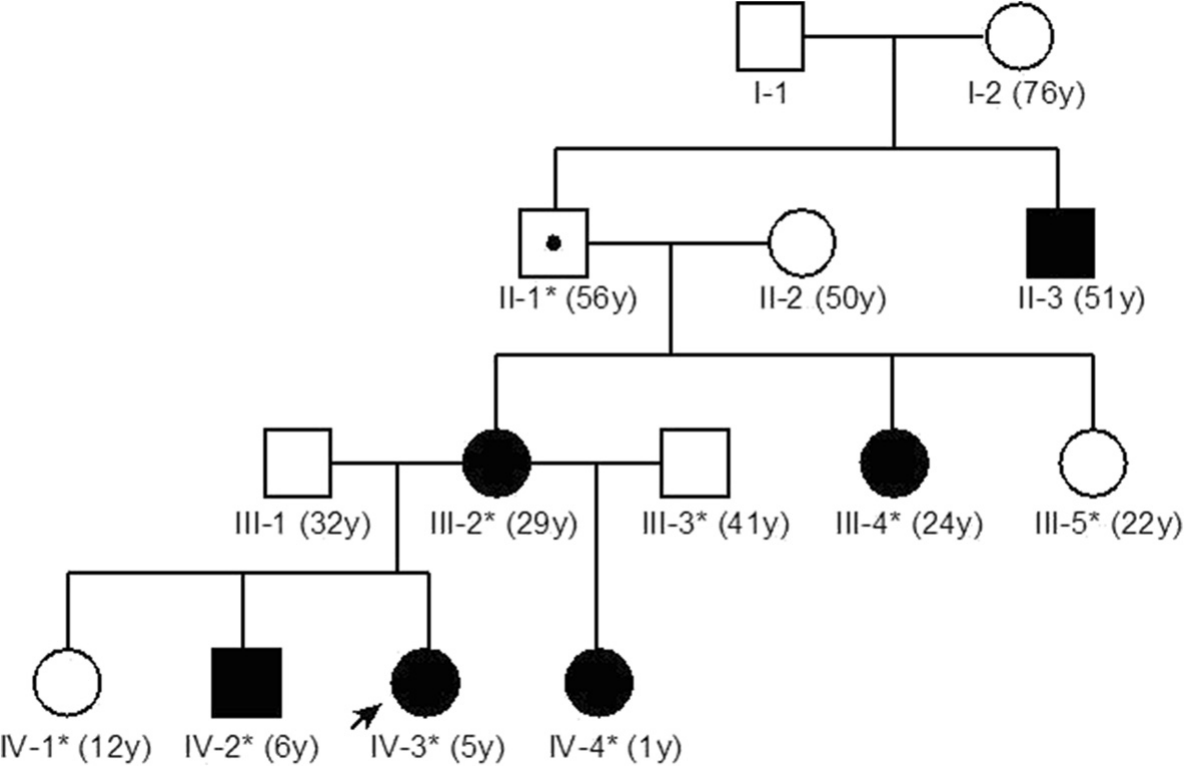
Familial pedigree. Autosomal-dominant inheritance is suspected. y, years. For each family member, age at the time Patient 1 first presented for medical attention is shown.

**Figure 2:**
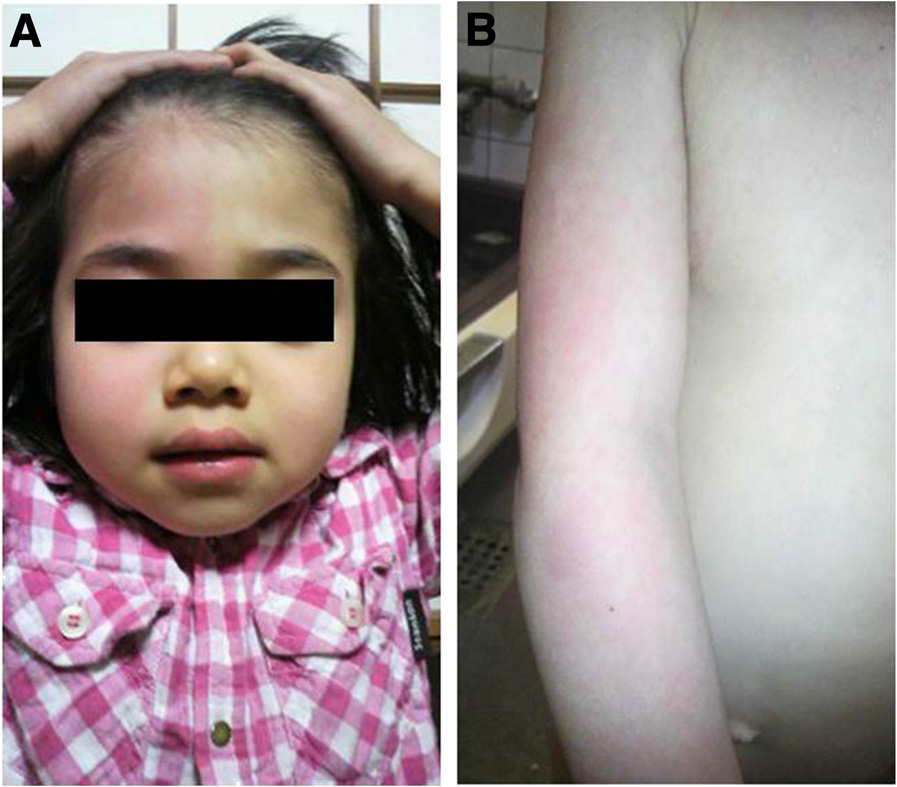
Clinical findings. Flushing of **(A)** the face and **(B)** arm after a right-sided severe headache attack in Patient 1.

The index patient (Patient 1: IV-3 in Figure 1) was a 5-year-old girl with a history of recurrent, unilateral temporal headaches lasting 20–90 s associated with ipsilateral conjunctival injection, lacrimation, nasal congestion, rhinorrhea, and facial flushing. Headaches were often triggered by hitting her head or body, taking a bath, experiencing a temperature change, or sleeping. Her past medical history included a febrile convulsion at 15 months old. She experienced the first episode of severe headache with thrashing and crying out at 16 months old. Thereafter, the frequency of moderate or severe headache attacks gradually increased to 10–20 times per day. Attacks initially emerged during sleep, but later predominantly occurred during the daytime while the patient was awake. When experiencing a severe attack, she screamed and thrashed, and bilateral lacrimation and facial flushing occurred (Additional file 1: Video S1). Unilateral, mainly right-sided, facial and upper-extremity flushing (Figure 2) appeared 5–10 min after severe attacks and lasted from half an hour to a few hours. She had undergone repeated medical examinations, such as routine laboratory investigations, brain computed tomography, magnetic resonance imaging (MRI), and electroencephalography (EEG), but no organic abnormalities had been identified, and she had received no medical treatment.

When the patient sought medical attention from our institution, we tentatively diagnosed short-lasting unilateral neuralgiform headache attacks with cranial autonomic symptoms (SUNA), in accordance with the criteria of the International Classification of Headache Disorders [11]. After initiation of treatment with carbamazepine at 50 mg/day (3.125 mg/kg/day) at bedtime, the frequency and intensity of attacks decreased within a couple of weeks, and no adverse drug reactions were seen. An increase in the carbamazepine dosage to 100 mg/day resulted in complete disappearance of the attacks within 2 weeks. When her parents forgot to give her the carbamazepine, the attacks began again.

Family history revealed similar headaches with autonomic symptoms in an older brother (Patient 3: IV-2), a younger sister (Patient 2: IV-4), her mother (III-2), her maternal aunt (III-4), and her maternal grandfather’s brother (II-3). The headache attacks of her mother, maternal aunt, and maternal grandfather’s brother all showed spontaneous remission in adulthood. The aunt has experienced episodic burning pain in the legs since adolescence. The maternal grandfather had suffered epilepsy and headache attacks with conjunctival injection. Autosomal-dominant inheritance of this disease was inferred from the pedigree (Figure 1).

### Patient 2 (IV-4)

Headaches with similar features to those in her sister emerged at 18 months old and were accompanied by hemi-facial flushing after the severe headache attacks. She had the same triggers as her sister, and in addition, her headaches were often precipitated by defecation and associated with external genital pain. She was treated with carbamazepine at 35 mg/day (3.2 mg/kg/day at bedtime), but this treatment showed little effect. After the dosage of carbamazepine was increased to 50 mg/day, the frequency of attacks decreased from 10–20 times per day to 1–2 times per day.

### Patient 3 (IV-2)

Headaches had appeared at 2 years old and were accompanied by hemi-facial flushing after severe attacks. At 3 years old, headaches sometimes occurred during defecation, along with enlargement and pain of the penis. At 5 years old, the frequency of headaches had increased to 1–2 times per week. The patient’s mother did not remember the frequency of headaches before 5 years old, because his headache attacks were less frequent and severe than those of the proband. His headache attacks were able to be prevented with carbamazepine at 100 mg/day.

### Patient 4 (III-2)

She had experienced moderate to severe unilateral headaches since childhood, associated with ipsilateral conjunctival injection and flushing that lasted 1–2 min several times a year. Headaches occurred frequently around 3 to 5 years old, similar to those in her children. In addition to headache attacks, moderate burning pain in her legs occasionally occurred during defecation. She also had scoliosis.

### Patient 5 (III-4)

She had experienced moderate unilateral headaches with ipsilateral conjunctival injection since childhood. Genital pain sometimes occurred during defecation, with enlargement of the external genitalia. She had chronic pain in her extremities that emerged in childhood and worsened in the lower limbs when she was a high school student. Severe pain was occasionally provoked by light touch and warming, and was relieved by cooling.

### Patient 6 (II-3)

Patient 6, a younger brother of the maternal grandfather of Patients 1–3, had moderate to severe unilateral headaches with ipsilateral conjunctival injection in childhood. He did not remember any further detailed features concerning the headaches.

## Methods

In an attempt to identify the background condition of the painful disorder and to find any structural or functional abnormalities in the central and peripheral nervous systems of the family members, Patients 1–3 were admitted for further evaluation. We observed and recorded all headache attacks of the three patients in the hospital for 3 days. We also performed routine laboratory examination, spinal radiography, brain MRI, brain magnetic resonance angiography, and nerve conduction studies in all patients. Patients 1 and 2 underwent a 3-day period of video-EEG monitoring. Patient 3 underwent routine EEG, as well as examination of somatosensory evoked potentials. Brain MRI examinations were performed with 3.0-T scanners (Siemens Magnetom Verio, Munich, Berlin and Erlangen, Germany: Patients 1 and 3; Philips Achieva, Amsterdam, the Netherlands: Patient 2). Cervical and thoracic MRI was performed in Patient 2. We conducted interictal single photon emission computed tomography (SPECT) scans with ^99m^Tc-ethyl cysteinate dimer in Patients 1 and 2. Ictal SPECT of Patient 2, whose headaches were the most frequent among the siblings, was also successfully performed. The subtraction ictal SPECT co-registered to MRI (SISCOM), which improves the sensitivity and specificity of SPECT under paroxysmal conditions [12,13], was performed using a previously reported method [14]. To identify the genetic causation in this pedigree, WES and WES-based linkage analysis were performed using DNA from nine family members (the individuals with asterisks in Figure 1: six affected and three unaffected family members) based on an autosomal-dominant model of inheritance. Detailed methods are described in the Supplementary methods. We considered that Patient II-1 possessed a heterozygous mutation with incomplete penetrance.

### Standard protocol approvals, registrations, and patient consent

This study conformed to the ethical standards described in the Declaration of Helsinki and was approved by the institutional review boards at both Japanese Red Cross Shizuoka Hospital and Yokohama City University School of Medicine. The parents of the patient 1, 2 and 3 provided written informed consent and the authorization to publish any photos or videos of recognizable persons.

## Results

Upon examination, general and neurological findings were unremarkable other than a tendency toward hyporeflexia in the three subjects. The clinical features of these patients are summarized in Table 1.

**Table 1:** **Clinical findings in our study of three cases with short-lasting trigeminal autonomic cephalalgia**

	**Patient 1,**	**Patient 2,**	**Patient 3,**
	**Female**	**Female**	**Male**
Severity of the pain	Moderate or severe	Moderate or severe	Mild to severe
Location of the pain	Unilateral, mainly left-sided forehead, temple and retro-orbital region	Unilateral forehead, temple and retro-orbital region	At the beginning bilateral orbital pain, immediately changed to ipsilateral frontal and temporal headache, mainly right-sided
Duration of attacks	20-90 s	20-90 s	About 1 min
Frequency of attacks during the 3 days of admission	19 times; 17 times during sleep, and four times during the daytime nap.	19 times	3 times
Ipsilateral autonomic features			
Conjunctival injection	+	?*	**-**
Lacrimation	+	+	**-†**
Nasal congestion	+	+	**-**
Rhinorrhea	+	+	**-**
Eyelid edema	**-**	-	+‡
Forehead and facial sweating	**-**	-	**-**
Forehead and facial flushing	+	+	+
Sensation of fullness in the ear	**-**	**-**	**-**
Miosis	**-**	**-**	**-∏**
Ptosis	**-**	**-**	**-**
Agitation	+	+	+
Premonitory features or auras	None	None	Uncomfortable feeling

*We could not observe this feature because of tight closing of her eyes.

†We observed bilateral lacrimation.

‡We observed hemifacial edema.

∏ We observed contralateral miosis one time.

**Figure 3:**
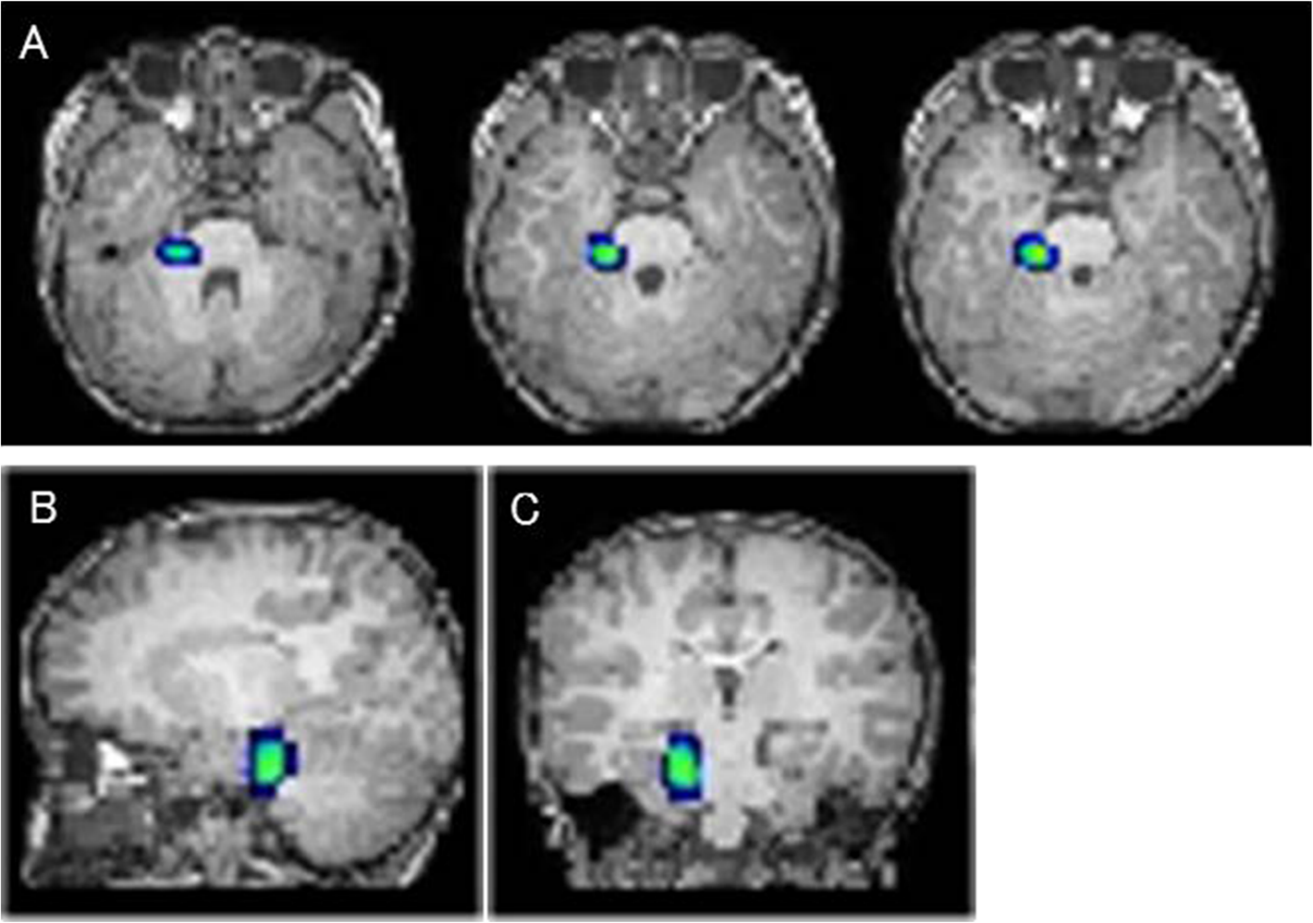
SPECT co-registered to MRI (SISCOM) analysis in Patient 2. **A)** Transverse, **B)** sagittal, and **C)** coronal images. A focal increase in perfusion is noted at the right trigeminal root entry zone during a right-sided attack.

Video-EEG monitoring recorded 19 attacks each in Patients 1 and 2 and demonstrated no ictal EEG changes during the headache attacks. Spinal X-rays revealed scoliosis in Patient 3, but not in Patients 1 and 2. All patients showed normal results from nerve conduction studies. Routine laboratory examination, head MRI, and head magnetic resonance angiography were normal in all cases. In Patient 1, MRI of the cervical and thoracic spine was performed in an attempt to identify sympathetic nerve lesions leading to facial flushing, but yielded normal results. Unexpectedly, stimulation of the median nerves (5 Hz, 500 counts) to assess somatosensory evoked potentials provoked left-sided temporal headache attacks in Patient 3. SISCOM analysis demonstrated focal hyperperfusion of the right trigeminal root entry zone during a right-sided attack in Patient 2 (Figure 3). Interictal SPECT images from Patient 1 were unremarkable.

**Figure 4:**
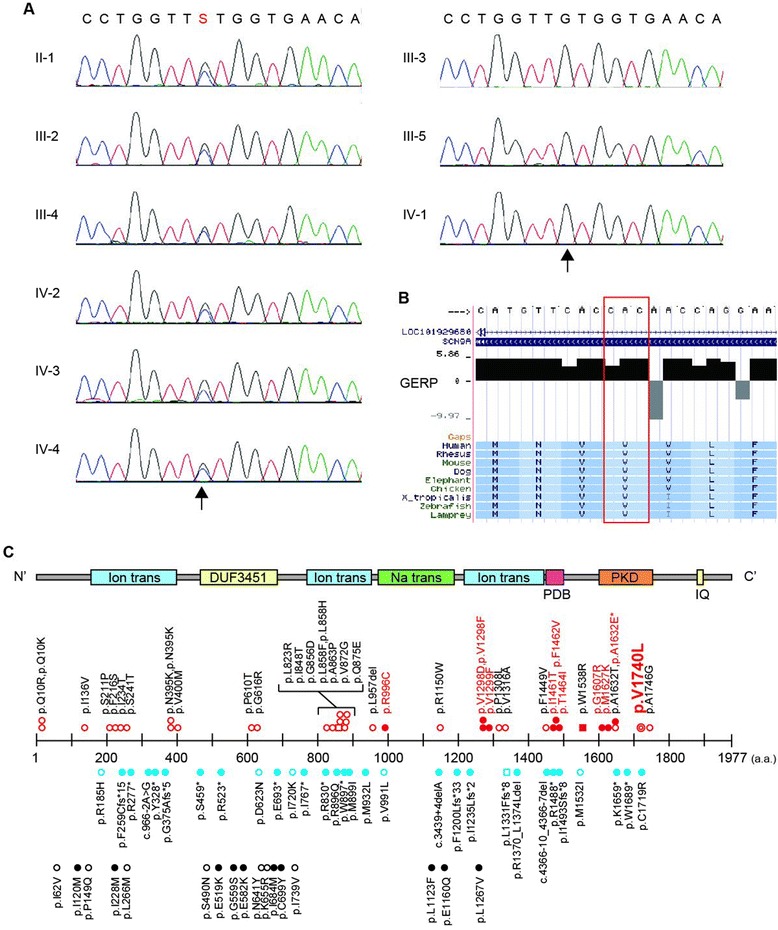
Genetic analysis of the pedigree. Electropherograms of the heterozygous *SCN9A* mutation. Arrow indicates the position of the mutation (NM_002977: c.5218G>C, p.Val1740Leu). Data from six affected and three unaffected individuals are shown on the left and right panels, respectively. A G/C change is represented as an “S” according to the IUPAC code. **B)** Evolutionary conservation of p.Val1740 in *SCN9A* from lampreys to humans. The altered amino acid residue is marked with a red box. The degree of evolutionary conservation was scored by Genomic Evolutionary Rate Profiling. **C)** The upper portion illustrates the SCN9A protein with functional domains predicted by the SMART program using the protein sequence (NP_002968). Ion trans, ion transport domain; DUF3451, DUF3451 domain; Na trans, sodium ion transport-associated domain; PDB, PDB domain; PKD, PKD (polycystic kidney) channel domain; IQ, IQ motif (calmodulin-binding motif). Middle and lower portions indicate the location of human *SCN9A* mutations: red double circles, mutation identified in this pedigree; red open circles, erythermalgia; red filled circles, PEPD; red open squares, pain; dysautonomia and acromesomelia; red filled squares, chronic non-paroxysmal neuropathic pain; blue open circles, small fiber neuropathy; blue open square, hereditary sensory and autonomic neuropathy type IID; blue filled circles, indifference to pain; black open circles, febrile convulsion; black filled circles, Dravet syndrome. *The patient with p.A1632E showed both erythromelalgia and PEPD [21].

**Figure 5:**
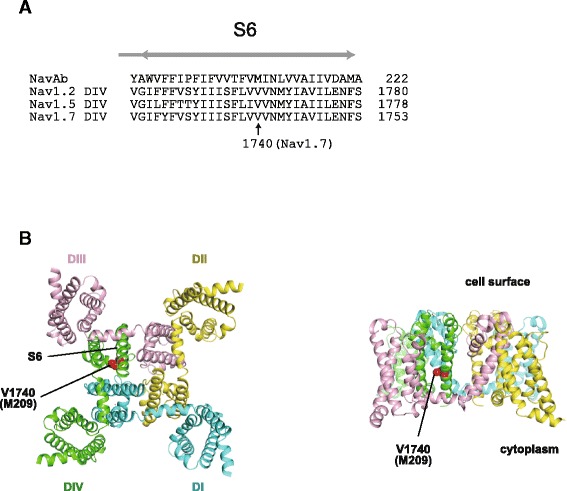
Structural implications of the mutated *SCN9A* gene product. **A)** Multiple alignment of amino acid sequences from the S6 transmembrane segments in domain IV from human Na_v_1.2, 1.5, and 1.7 with the S6 pore-lining segment from NavAb [15]. **B)** Crystal structure of a voltage-gated sodium channel from *Arcobacter butzleri* (NavAb) (PDB code 3rvz) [15] viewed from the intracellular side of the membrane (left) and from the side (right), with the identified mutant position indicated.

WES identified a heterozygous missense mutation (c.5218G>C, p.Val1740Leu) in *SCN9A* (NM_002977) in all affected family members, but not in any of the unaffected members (Additional file 2: Tables S1 & S2). This variant was confirmed with Sanger sequencing (Figure 4A). *SCN9A* is located on chromosome 2 within the linked region spanning 7.3 Mb, and showed a maximum logarithm of the odds score of 1.805 (Additional file 2: Table S3, Additional file 2: Figure S1). The altered amino acid (p.Val1740) is highly evolutionally conserved from lampreys to humans (Figure 4B). Based on the SMART program (http://smart.embl-heidelberg.de/) and UniProt KB (http://www.uniprot.org/uniprot/), p.Val1740 was located in the transmembrane region of the polycystic kidney channel domain (Figure 4C, Additional file 2: Figure S2). This missense mutation was predicted to be pathogenic according to the *in silico* programs SIFT: 0, Polyphen-2: 0.883, and MutationTaster: 0.97471. Next, we evaluated the effects of the mutation on protein structures at the molecular structural level using an *in silico* model [15]. The human sodium channel Na_v_1.7 encoded by *SCN9A* consists of a long polypeptide that folds into four homologous domains (domains I-IV). Because the overall architecture of Na_v_1.7 and other voltage-gated ion channels is considered to be similar, we mapped the identified mutation on the available high-resolution structure of a sodium channel from *Arcobacter butzleri* (NavAb) (PDB code 3rvz) (Figure 5). Val 1740, which corresponds to Met 209 of NavAb as determined from amino acid alignment, is predicted to be located within the S6 segment of domain IV and in the hydrophobic core, suggesting a potential impact of the Val1740Leu mutation on Na_v_1.7 function.

## Discussion

Patient 1 was initially diagnosed with SUNA based on the symptoms of unilateral temporal headaches lasting 20–90 s in association with cranial autonomic symptoms. However, harlequin-type flushing and pain of the external genitalia accompanied headache in some of her relatives, representing a quite unusual occurrence in SUNA. The p.Val1740Leu mutation in *SCN9A*, identified in all affected family members, but not in any unaffected members, suggested that the clinical condition in this family may be regarded as a novel variant of PEPD. Although headaches not localized to the orbital and submaxillary areas have not been reported in PEPD, the unusual aspects of headache-associated phenomena were reminiscent of those in PEPD.

SISCOM findings from a patient in the present family support the idea that augmented excitation of the trigeminal nerve is essential for the emergence of headaches in certain cases of short-lasting, unilateral headaches. Functional MRI studies have revealed functional changes in the nervous systems accompanying similar short-lasting, unilateral headaches in the trigeminal areas as follows: activation of the red nucleus, ventral pons, trigeminal root entry zone, and hypothalamus ipsilateral to the pain side in subjects with cluster headache [16], activation in the region of the ipsilateral hypothalamic gray matter in short-lasting unilateral neuralgiform headache attacks with conjunctival injection and tearing (SUNCT) [17], and bilateral hypothalamic activation during the pain attacks in a SUNCT patient [18]. This last patient became completely pain-free after surgical decompression of the ipsilateral trigeminal nerve. We hypothesized that a peripheral trigger with ectopic excitation may have contributed to the clinical picture of SUNCT in this patient. In addition, SPECT images of two patients with SUNA showed normal tracer uptake and symmetric perfusion during headache episodes [19]. The hyperperfusion observed at the trigeminal root entry zone in this study may support the trigeminovascular theory for the pathogenesis of migraines and trigeminal autonomic cephalalgia, assuming a primary pathological activation of trigeminal ganglion cells and resultant antidromic pain generation through neuropeptide release at the vessel walls. The provocation of headache by repetitive stimulation of somatosensory afferents is important, because cluster-like attacks can be provoked in cluster headache patients by experimental low-frequency stimulation of the sphenopalatine ganglion at 5 Hz [20]. This phenomenon may be related to aberrant Na_v_1.7 function, and the possibility of a role for mutant *SCN9A* in the scheme of trigeminovascular theory requires further study.

Lastly, it is interesting that the mutations causing PEPD are mostly located on the C-terminal half of the SCN9 protein, as commonly seen in the present family, whereas those related to Dravet syndrome/febrile convulsions are located on the N-terminal half (Figure 4C). Differential impact from the site of *SCN9A* mutations on the function in peripheral pain control and excitation of cortical neurons is assumed.

## Conclusions

A novel *SCN9A* mutation was found in a pedigree in which affected family members suffered from short-lasting, severe, unilateral temporal headaches characterized by the precipitation of headache by defecation. Such headaches were associated with pain of the external genitalia, harlequin-type facial flushing after headache events, as well as paroxysmal, burning limb, pain in some subjects. The *SCN9A* mutation in this family suggests that the phenotype of the patients represents an unusual variant of PEPD. This report expands on the clinical spectrum of *SCN9A*-related painful disorders, and supports the essential role of aberrant peripheral activation in the pathogenesis of trigeminal nerve-related, short-lasting, primary headaches.

## Additional files

Additional file 1:
**Video S1.** Headache attack of Patient 2. The video was taken by the patient’s mother just after the beginning of a severe headache attack. The patient screams and thrashes, and bilateral lacrimation and facial flushing are observed. The attack lasted for 1 minute. Additional file 2:
**Methods, linkage analysis, tables and figures.**

## Abbreviations

PEPD: Paroxysmal extreme pain disorder
WES: Whole-exome sequencing
MRI: Magnetic resonance imaging
EEG: Electroencephalography
SUNA: Short-lasting unilateral neuralgiform headache attacks with cranial autonomic symptoms
SPECT: Single photon emission computed tomography
SISCOM: Subtraction ictal SPECT co-registered to MRI
