# Supplementary material for: Short-lasting unilateral neuralgiform headache attacks with ispilateral facial flushing is a new variant of paroxysmal extreme pain disorder
Source: J Headache Pain. 2015 Apr 23;16:35. doi: 10.1186/s10194-015-0519-3 (PMC4414864; doi:10.1186/s10194-015-0519-3)
Supplement: Additional file 1: — Video S1. Headache attack of Patient 2. The video was taken by the patient’s mother just after the beginning of a severe headache attack. The patient screams and thrashes, and bilateral lacrimation and facial flushing are observed. The attack lasted for 1 minute. [file 10194_2015_519_MOESM1_ESM.docx]

Video legend

Video 1. Headache attack of Patient 2. The video was taken by the patient’s mother just after the beginning of a severe headache attack. The patient screams and thrashes, and bilateral lacrimation and facial flushing are observed. The attack lasted for 1 minute.
