# Supplementary material for: Short-lasting unilateral neuralgiform headache attacks with ispilateral facial flushing is a new variant of paroxysmal extreme pain disorder
Source: J Headache Pain. 2015 Apr 23;16:35. doi: 10.1186/s10194-015-0519-3 (PMC4414864; doi:10.1186/s10194-015-0519-3)
Supplement: Additional file 2: — Methods, linkage analysis, tables and figures. [file 10194_2015_519_MOESM2_ESM.doc]

**Supplementary Methods**

Whole exome sequencing was performed as previously reported [e1] with some modifications. In brief, 3 μg genomic DNA was sheared with sonication using Covaris S2 (Covaris Inc., Woburn, MA) according to the manufacturer’s instructions. The coding region was enriched using a SureSelect Human All Exon V5 kit (Agilent Technologies, Santa Clara, CA) and read using HiSeq2000 with 101-bp paired-end reads and seven indices (Illumina, San Diego, CA). Reads were aligned to human genome hg19 with Novoalign 3.00.02 ([http://www.novocraft.com](http://www.novocraft.com/)). After PCR duplication with Picard (<http://picard.sourceforge.net/>), variants were called with Genome Analysis Toolkit 2.7-4 (GATK: http://www.broadinstitute.org/gatk/) and annotated with ANNOVAR (1000 Genomes Project 2013 June release) (<http://www.openbioinformatics.org/annovar/>). Using this in silico flow, the common variants (minor allele frequency ≥1%) registered in dbSNP135 (<http://www.ncbi.nlm.nih.gov/projects/SNP/snp_summary.cgi?view+summary=view+summary&build_id=135>) were filtered out. Using genotyping calling, candidate variants within the coding region and the adjacent ±30 bp were selected as follows: 1) variants shared by affected individuals, 2) variants that were not observed in all unaffected individuals, 3) variants that were not registered in ESP6500 (<http://evs.gs.washington.edu/EVS/>), HGVD (<http://www.genome.med.kyoto-u.ac.jp/SnpDB/about.html>), or dbSNP137 ( <http://www.ncbi.nlm.nih.gov/projects/SNP/snp_summary.cgi?view+summary=view+summary&build_id=137>), 4) variants that were not observed in our in-house Japanese control cohort (n = 575), 5) nonsynonymous variants.

**Linkage analysis**

The calibrated bam files (the intermediate file derived from exome sequencing) of each sample were used for variant calling by samtools. Informative SNPs were selected by Linkdatagen [e2] to obtain the call file (brlmm). Using this call file, linkage analysis (parametric multipoint analysis) was performed to calculate the logarithm of the odds (LOD) score using Allegro version2 [e3]. Because autosomal dominant inheritance was considered, we focused on linked regions with a maximum LOD score ≥1.8 because six affected individuals were included in this analysis.

**Supplementary Figure legends**

**Supplementary Figure e-1. LOD scores from the linkage analysis**

LOD scores were calculated for each chromosome using Allegro version 2. X and Y axes indicate the genomic position of the chromosome (p terminal (left) to q terminal (right)) and the LOD scores.

**Supplementary Figure e-2. Localization and evolutionary conservation of the mutation.** p.Val1740 is localized at the transmembrane region. Using UniProtKB, transmembrane regions are shown using isoform 1 (protein length = 1,988 amino acids). Using CLUSTAL 2.1 multiple sequence aligner (<http://clustalw.ddbj.nig.ac.jp/index.php?lang=ja>), the transmembrane region in NP_002968 is highlighted in the yellow box. The altered amino acid residue p.Val1740 is written in red.

**Supplementary Figure e-1.** LOD score of the SUNA pedigree

**
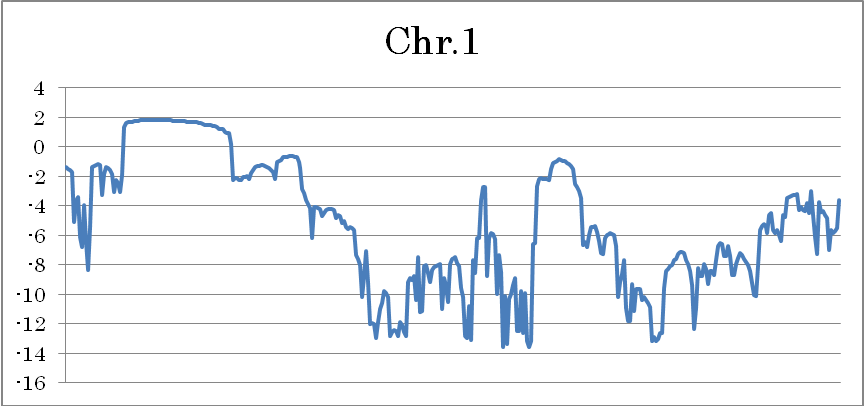

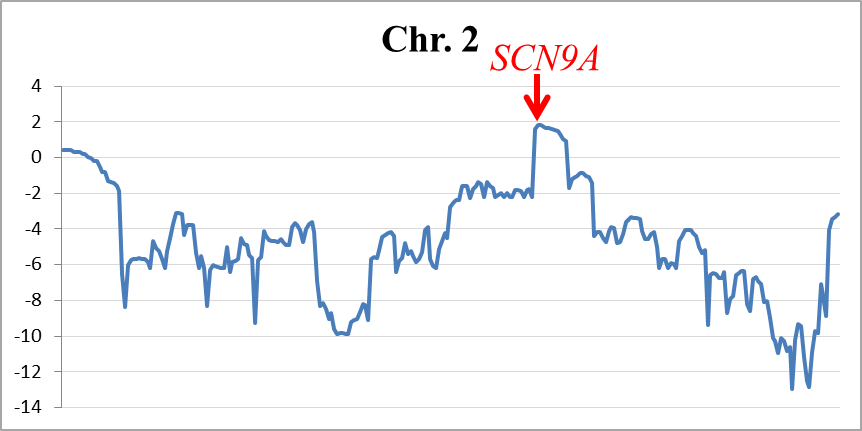
**

**
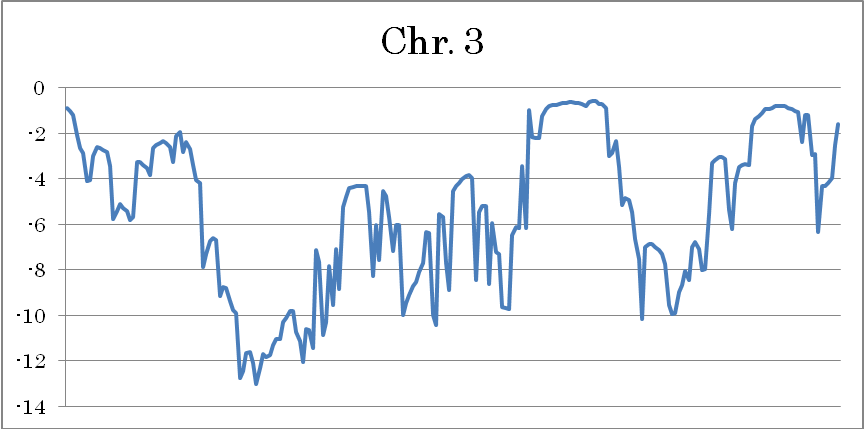
**

**
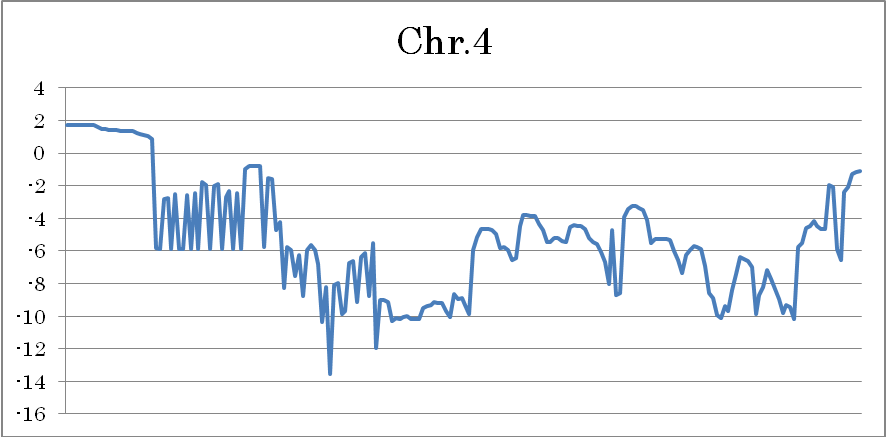
**

**
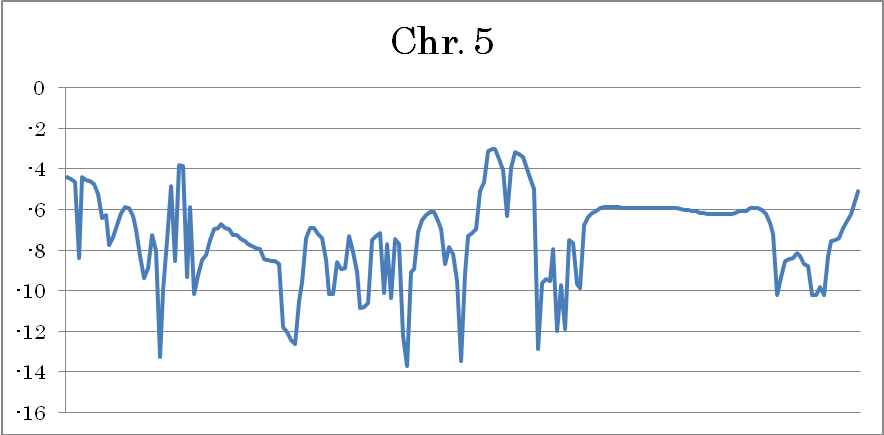
**

**
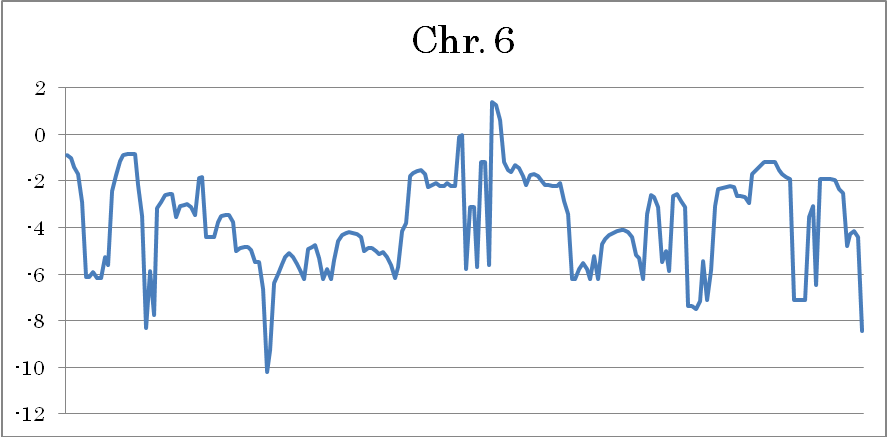
**

**
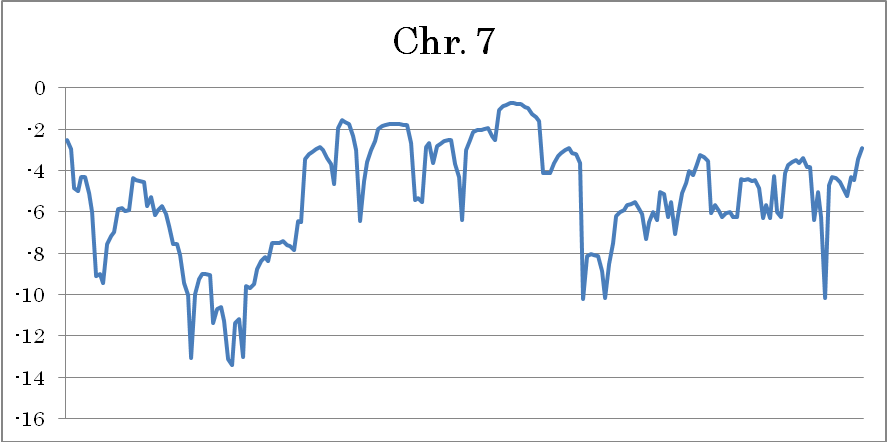
**

**
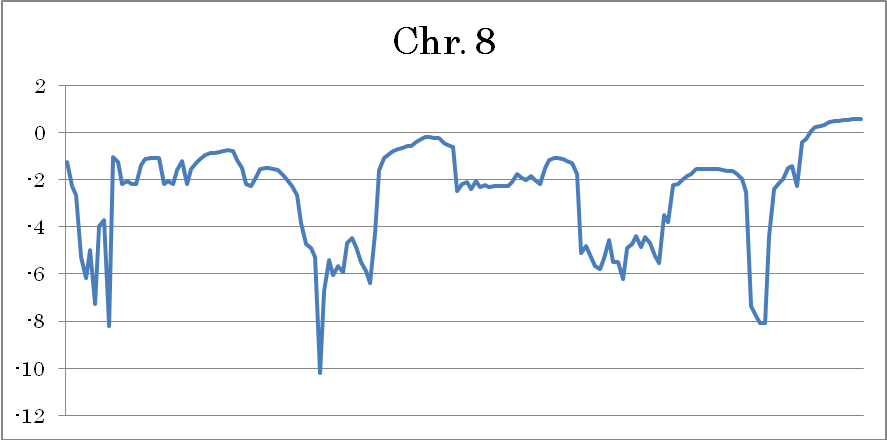
**

**
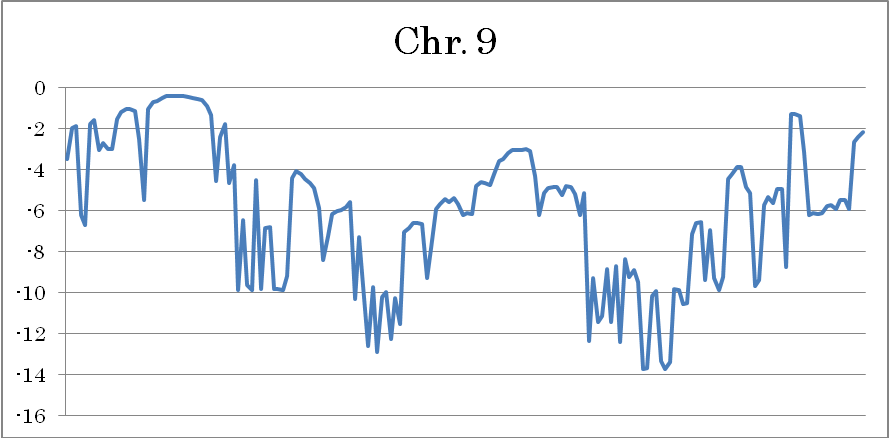
**

**
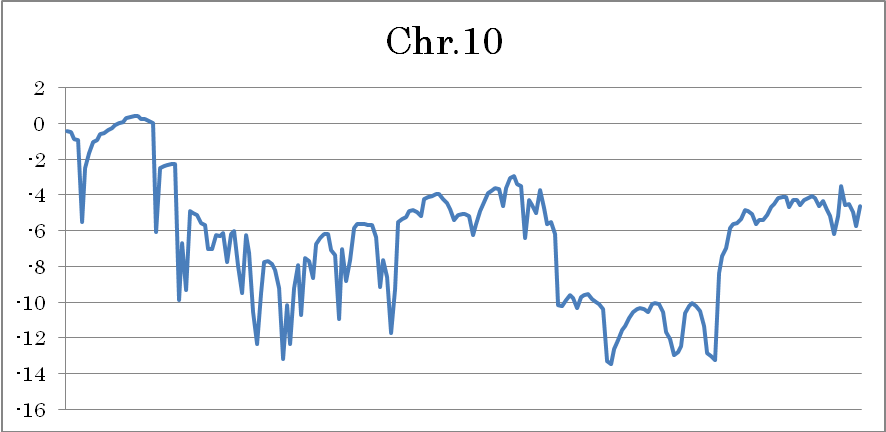
**

**
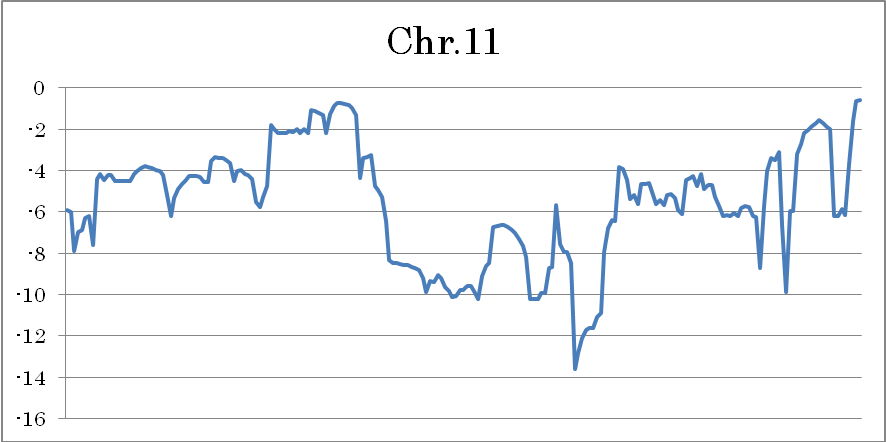
**

**
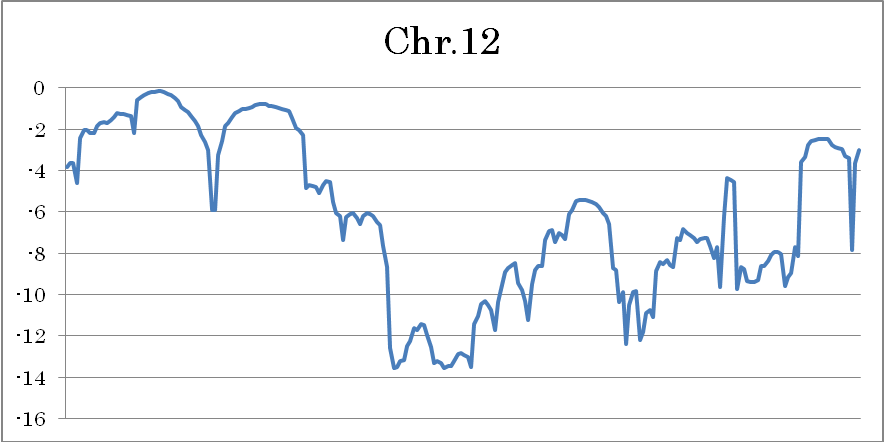
**

**
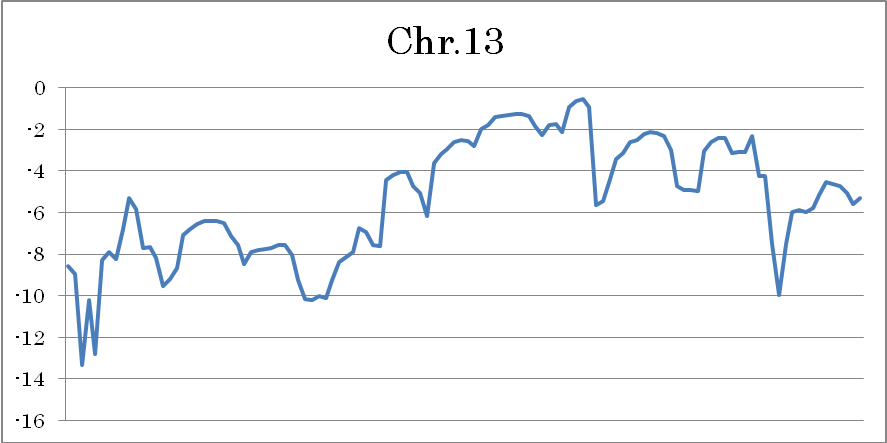
**

**
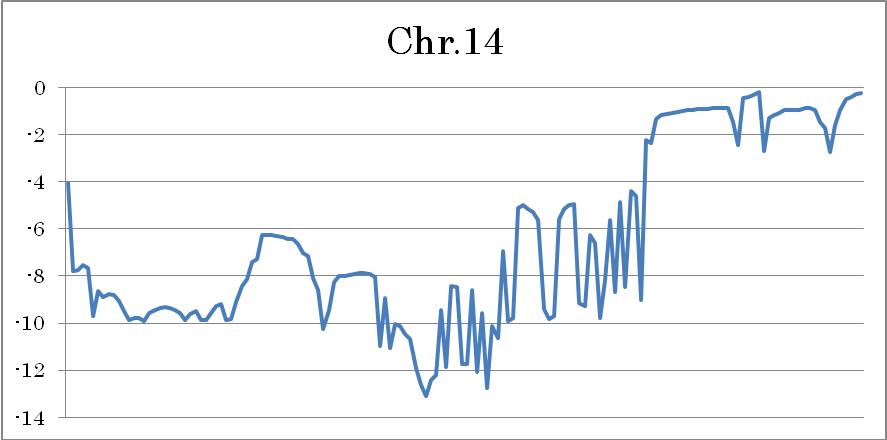
**

**
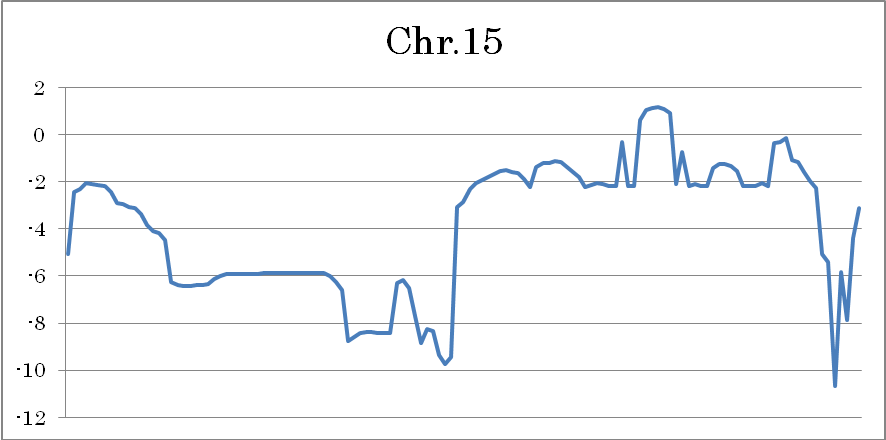
**

**
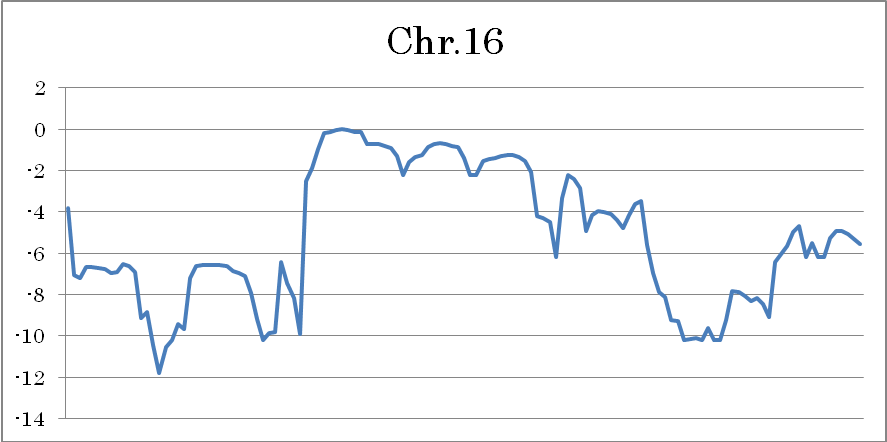
**

**
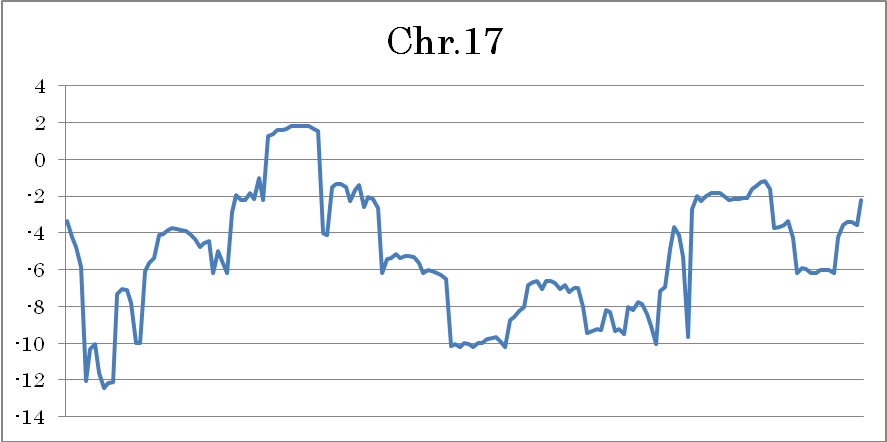
**

**
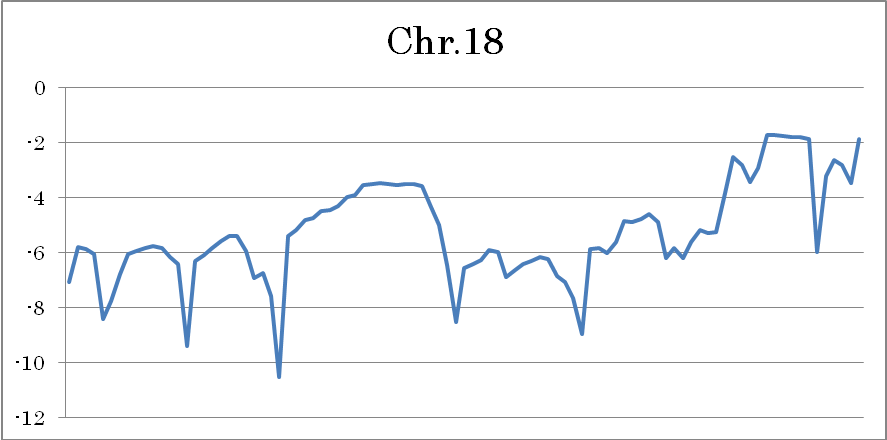
**

**
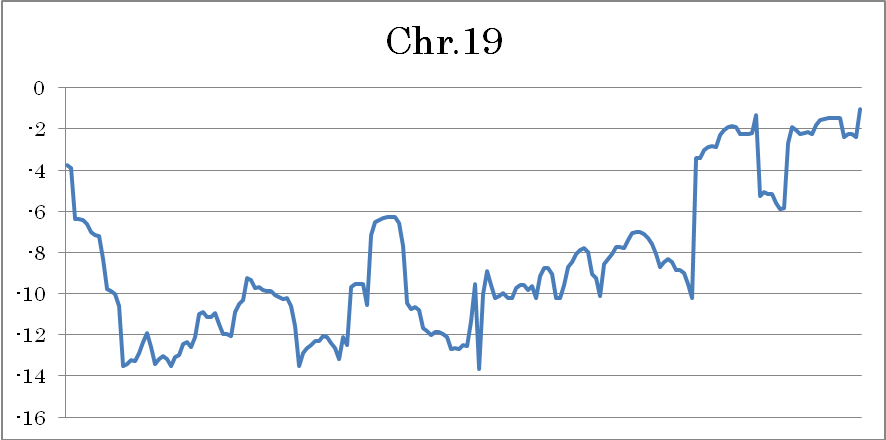
**

**
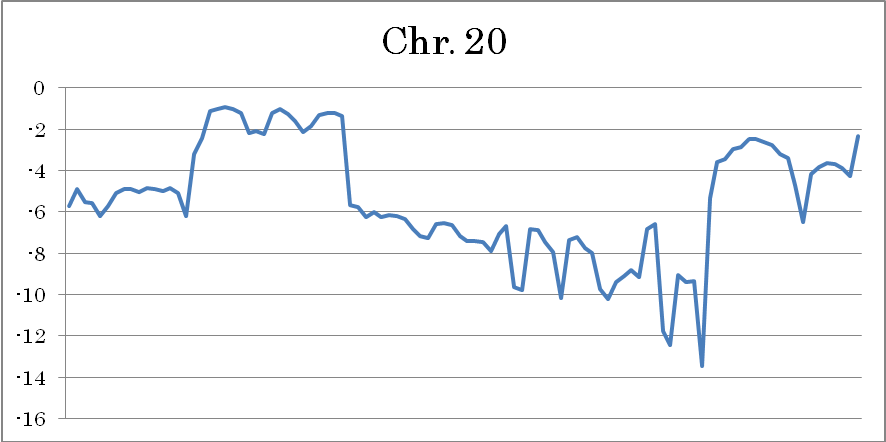

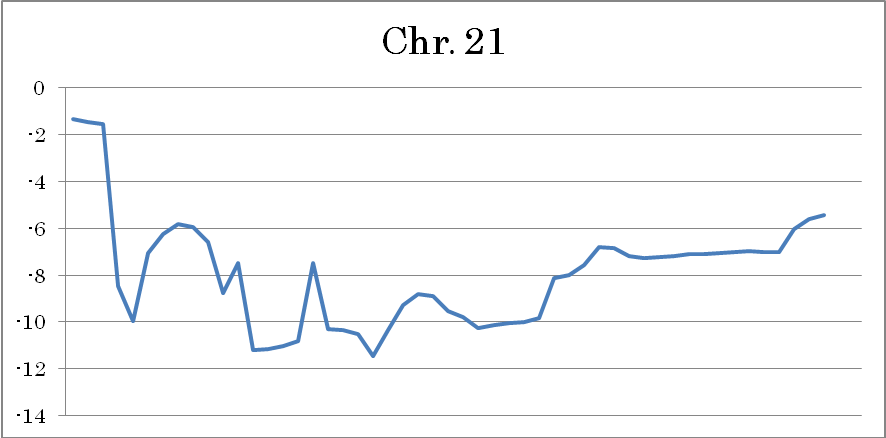
**

**
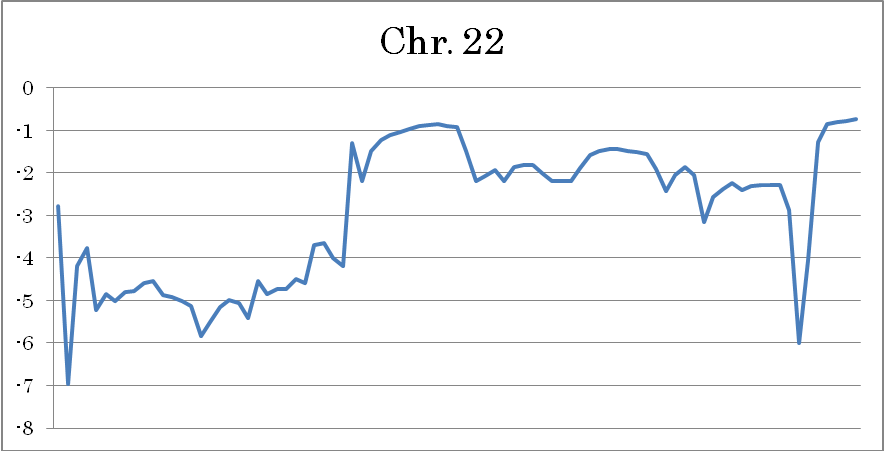
**

**Supplementary Figure e-2.** Location of Valine1740 within the transmembrane region

sp|Q15858|SCN9A_HUMAN_iso1 DGLLAPILNSKPPDCDPKKVHPGSSVEGDCGNPSVGIFYFVSYIIISFLV 1750

sp|Q15858-2|SCN9A_HUMAN_iso2 DGLLAPILNSKPPDCDPKKVHPGSSVEGDCGNPSVGIFYFVSYIIISFLV 1750

sp|Q15858-3|SCN9A_HUMAN_iso3 DGLLAPILNSKPPDCDPKKVHPGSSVEGDCGNPSVGIFYFVSYIIISFLV 1739

NP_002968 DGLLAPILNSKPPDCDPKKVHPGSSVEGDCGNPSVGIFYFVSYIIISFLV 1739

**************************************************

sp|Q15858|SCN9A_HUMAN_iso1 **V**VNMYIAVILENFSVATEESTEPLSEDDFEMFYEVWEKFDPDATQFIEFS 1800

sp|Q15858-2|SCN9A_HUMAN_iso2 **V**VNMYIAVILENFSVATEESTEPLSEDDFEMFYEVWEKFDPDATQFIEFS 1800

sp|Q15858-3|SCN9A_HUMAN_iso3 **V**VNMYIAVILENFSVATEESTEPLSEDDFEMFYEVWEKFDPDATQFIEFS 1789

NP_002968 **V**VNMYIAVILENFSVATEESTEPLSEDDFEMFYEVWEKFDPDATQFIEFS 1789

**************************************************

**Supplementary Table e-**1

| Filter | Number of the variants |
| --- | --- |
| Total variants | 12559 |
| Variants sheared by Affected individual | 3597 |
| Variants not sheared by unaffected individual | 21 |
| Not registered in ESP6500 | 13 |
| Not registered in HGVD | 5 |
| Not registered in dbSNP137 | 4 |
| Not registered in In-house exome (n = 575) | 2 |
| Remove synonymous variants | 1 |

**Supplementary Table e-**2

| DNA ID | Sample ID | Affection status | PCR duplication | Mean depth | % above x5 | % above x10 |
| --- | --- | --- | --- | --- | --- | --- |
| 9408 | II-1 | carrier | 0.040112 | 73.83 | 96.9 | 95.3 |
| 8712 | III-2 | affected | 0.03175 | 91.28 | 97.1 | 96 |
| 8711 | III-3 | unaffected | 0.025553 | 57.07 | 96.5 | 94 |
| 9409 | III-4 | affected | 0.026666 | 99.7 | 97.2 | 96.2 |
| 9410 | III-5 | unaffected | 0.02868 | 91.99 | 97.1 | 96 |
| 9407 | IV-1 | unaffected | 0.033588 | 80.2 | 96.8 | 95.4 |
| 8708 | IV-2 | affected | 0.03907 | 96.63 | 97.2 | 96.2 |
| 8709 | IV-3 | affected | 0.034457 | 97.34 | 97.2 | 96.2 |
| 8710 | IV-4 | affected | 0.033923 | 90.41 | 97.1 | 96 |

**Supplementary Table e-**3

| Chromosome | Start | End | Distance (Mb) | Max. LOD |
| --- | --- | --- | --- | --- |
| 1 | 12776344 | 40431727 | 27.66 | 1.8057 |
| 2 | 166012203 | 173337495 | 7.33 | 1.805 |
| 17 | 12799985 | 21535937 | 8.74 | 1.8059 |

The physical position is based on hg19. *SCN9A* is located in chromosome 2 region 167051697-167232497.

**e-REFERENCES**

e1. Nakajima J, Okamoto N, Tohyama J, Kato M, Arai H, Funahashi O, Tsurusaki Y, Nakashima M, Kawashima H, Saitsu H, Matsumoto N, Miyake N (2014) De novo EEF1A2 mutations in patients with characteristic facial features, intellectual disability, and autistic behaviors and epilepsy. Clin Genet. doi: 10.1111/cge.12394.

e2. Bahlo M, Bromhead CJ (2009) Generating linkage mapping files from Affymetrix SNP chip data. Bioinformatics 25:1961-1962.

e3. Gudbjartsson DF, Jonasson K, Frigge ML, Kong A. Allegro (2000) a new computer program for multipoint linkage analysis. Nat Genet 25:12-13.
